# Clinical presentation of post-COVID pain and its impact on quality of life in long COVID patients: a cross-sectional household survey of SARS-CoV-2 cases in Bangladesh

**DOI:** 10.1186/s12879-024-09267-3

**Published:** 2024-04-04

**Authors:** Md. Feroz Kabir, Khin Nyein Yin, Mohammad Saffree Jeffree, Fatimah Binti Ahmedy, Muhamad Faizal Zainudin, Ohnmar Htwe, Sharmila Jahan, Md. Zahid Hossain, K. M. Amran Hossain, Tofajjal Hossain, Iqbal Kabir Jahid, Sonjit Kumar Chakrovorty

**Affiliations:** 1 Department of Rehabilitation Medicine, Faculty of Medicine and Health Sciences, Universiti Malaysia Sabah, 88400 Kota Kinabalu, Sabah Malaysia; 2 Department of Physiotherapy and Rehabilitation, Jashore University of Science and Technology (JUST), Jashore, Bangladesh; 3 Faculty of Medicine, Universiti Teknologi MARA, Sungai Buloh, Malaysia; 4 Faculty of Medicine, University Kebangsaan Malaysia, Kuala Lumpur, Malaysia; 5 Department of Microbiology, Jashore University of Science and Technology (JUST), Jashore, Bangladesh; 6 Dhaka College of Physiotherapy, under the Faculty of Medicine, University of Dhaka, Dhaka, Bangladesh

**Keywords:** Long COVID, Pain, Quality of life, Bangladesh

## Abstract

**Background:**

Pain is one of the prevalent Long COVID Symptoms (LCS). Pain interferes with the quality of life (QoL) and induces disease burden.

**Purpose:**

The study aimed to elicit the clinical presentation of pain and determine the relationships between QoL and pain in LCS.

**Methods:**

This household cross-sectional study of 12,925 SARS-CoV-2 cases between July and December 2021 was carried out in eight administrative divisions of Bangladesh. Stratified random sampling from the cases retrieved from the Ministry of Health was employed. Symptom screening was performed through COVID-19 Yorkshire Rehabilitation Scale, and long COVID was diagnosed according to World Health Organization (WHO) criteria. The analyses were conducted using IBM SPSS (Version 20.00).

**Results:**

The prevalence of pain in long COVID was between 01 and 3.1% in the studied population. The study also found five categories of pain symptoms as LCS in Bangladesh: muscle pain 3.1% (95% CI; 2.4–3.8), chest pain 2.4% (95% CI; 1.8–3.1), joint pain 2.8% (95% CI; 2.2–2.3), headache 3.1% (95% CI; 2.4–3.8), and abdominal pain 0.3% (95% CI; 0.01–0.5). People with LCS as pain, multiple LCS, and longer duration of LCS had significantly lower quality of life across all domains of the WHOQOL-BREF (*P* < 0.001) compared to asymptomatic cases.

**Conclusion:**

Three out of ten people with long COVID experience painful symptoms, which can significantly reduce their quality of life. Comprehensive rehabilitation can improve the symptoms and reduce the burden of the disease.

**Supplementary Information:**

The online version contains supplementary material available at 10.1186/s12879-024-09267-3.

## Introduction

Long COVID is an emerging medical concern characterised by clinical symptoms that persist beyond 3 months from the onset of COVID-19 infection in which the symptoms last for at least 8 weeks [1]. These symptoms cannot be explained by any other clinical diagnosis, resulting in episodic disability for the affected individuals [2]. Globally, 43% of COVID-19 survivors report post-COVID-19 symptoms, with more than half of the people residing in Asia have post-COVID persistent symptoms [3]. In Bangladesh, studies estimate that between 16 and 24% of those recovering from COVID-19 infection face long COVID [2, 4]. The long COVID has a wide prevalence rate globally, ranging from 7 to 43% [2–4], and pain is one of the major long COVID symptoms [5].

Musculoskeletal complication is among the leading causes of pain in long COVID. According to a recent study conducted in Turkey, a staggering 89% of COVID-19 survivors reported experiencing at least one persistent symptom after 3 months. Of those individuals, 74.6% reported experiencing at least one rheumatic or musculoskeletal symptom. Even after 6 months, 59.6% of survivors experienced at least one symptom, with 43.2% reporting rheumatic or musculoskeletal symptoms. Notably, joint pain was reported by 18.6% of those with symptoms, and 15% reported myalgia after 6 months. The study also found that females are three times more likely than males to develop rheumatic or musculoskeletal symptoms [6]. A British study reported a prevalence of musculoskeletal pain ranging from 0.3 to 65.2% in long COVID, and the pain was localised to a particular region or generalised and widespread [7]. A study at the University of Cyprus found that up to 50% of people can suffer from chronic pain, and post-COVID-19 chronic pain affects 63.3% of patients. Women are at a higher risk of developing musculoskeletal symptoms such as neuropathic pain, myalgia, headache, and joint pain in long COVID [8].

Pain associated with long COVID is varied, encompassing generalised pain that can focus on different body areas such as the cervical and lumbar spine, lower extremities, and the joint line of upper extremities [9]. The nature of this pain can be both nociceptive and neuropathic [10]. The most common area for myalgia is the calf region, the arm, and the shoulder girdle [6]. The pain symptoms are relapsing and remitting. Interestingly, non-hospitalized patients report a higher prevalence of musculoskeletal complaints (14.7%) than those who were hospitalised (9.1%) [11]. Among hospitalised patients, those in the ICU reported a higher prevalence of persistent pain (33%) compared to those who did not require ICU admission (27%) [12]. There is no conclusive evidence on how the pain symptoms proceed in long COVID. The known pathophysiological mechanisms include a virus-induced prolonged inflammatory response that triggers inflammatory cytokines and provokes immune cell hyperactivation. Some studies also report activating angiotensin-converting enzyme-2 (ACE-2) [6, 12]. Furthermore, the pain experienced in long COVID has been linked to conditions like myalgic encephalomyelitis or chronic fatigue syndrome, adding to the complexity of its pathophysiology [13].

Long COVID imposes a significant degradation of QoL that proceeds towards a significant disease burden for the survivors [14]. Affected individuals show a marked decline in their health-related QoL, especially in the physical health domain, as assessed by the SF36 metrics [15]. The impaired QoL level depends upon the duration of suffering from the long COVID [15]. A study found that the negative effect of COVID-19 on QoL was associated with pain, anxiety, depression, and problems with mobility [16]. Studies suggest that pain symptoms cause bodily illness, impaired mobility, and prolonged suffering from biopsychosocial issues that can impair the QoL of long COVID patients [17].

In the Bangladeshi context, research on QoL and long COVID revealed a significant correlation between age, gender, and various health domains, including physical and psychological health, social relationships, and environmental health in the World Health Organization Quality of Life (WHOQOL-BREF) scale. A separate study showed a significant improvement in all aspects of the physical domain of quality of life [18] where persistent illness adversely affects all domain of quality of life [19]. Moreover, another study showed that though all the domains improved significantly over the period, the progress in the physical domain was delayed [20]. Furthermore, those enduring prolonged symptoms of long COVID also experience a decline in their QoL [21]. However, the novelty of the study is examining the relationship between QoL and pain-specific symptoms along with determining predictive factors of long COVID in Bangladesh. Therefore, the objectives of the study were 1) to investigate socio-economic and clinical symptoms of people having pain as a long COVID symptom, 2) to assess the QoL of the people having pain symptoms, and 3) to determine the predictive factors of impaired QoL in long COVID individuals experiencing pain symptoms.

## Material and methods

### Study design, samples, and sampling method

We conducted a cross-sectional household survey between July and December 2021 among the COVID-19 patients diagnosed through the real-time Polymerase Chain Reaction test (RT-PCR) in the Bangladeshi testing centres. From our database, 12,925 people were shortlisted as samples with a probability of long COVID as they were tested 12 weeks prior. The primary inclusion criteria were aged 18 years and above who displayed long COVID symptoms, as identified by the COVID-19 Yorkshire Rehabilitation Scale (C19-YRS) screening tool, and provided consent for the household survey. Out of this pool, 4300 individuals met the primary criteria, and among them, 2507 completed face-to-face data collection and examination by trained data collectors. The long COVID diagnosis was based on the criteria set by the WHO Working Group classification [1]. The criteria of long COVID diagnosis was 1) developing or continuation new symptoms after 12 weeks of COVID-19 infections, and 2) symptoms persist at last 2 months with no other clinical diagnosis [1]. We divided and shortlisted data from the Directorate General of Health Services (DGHS) database by COVID-19 RT-PCR confirmation date. Then we estimated division-wise home screening samples by phone interview. Thus, we maintain the study’s external generality. We accessed the database with Ministry of Health and Family Welfare clearance. The overall sampling process was stratified to ensure representative samples from each administrative division of Bangladesh. Each division was a stratum, so it represented the administrative constituency of Bangladesh. The sample size calculation was conducted with a 95% confidence interval, 1.26 inter-cluster correlation, 1.6 design effect, and 5% margin of error. The required number of samples was 18,00, and for safety reasons and representative sampling, we included all the 2507 people to complete the research. The calculation for data collection was performed by software named Epi Info version 7.2.0.

### Study procedure

**Fig. 1:**
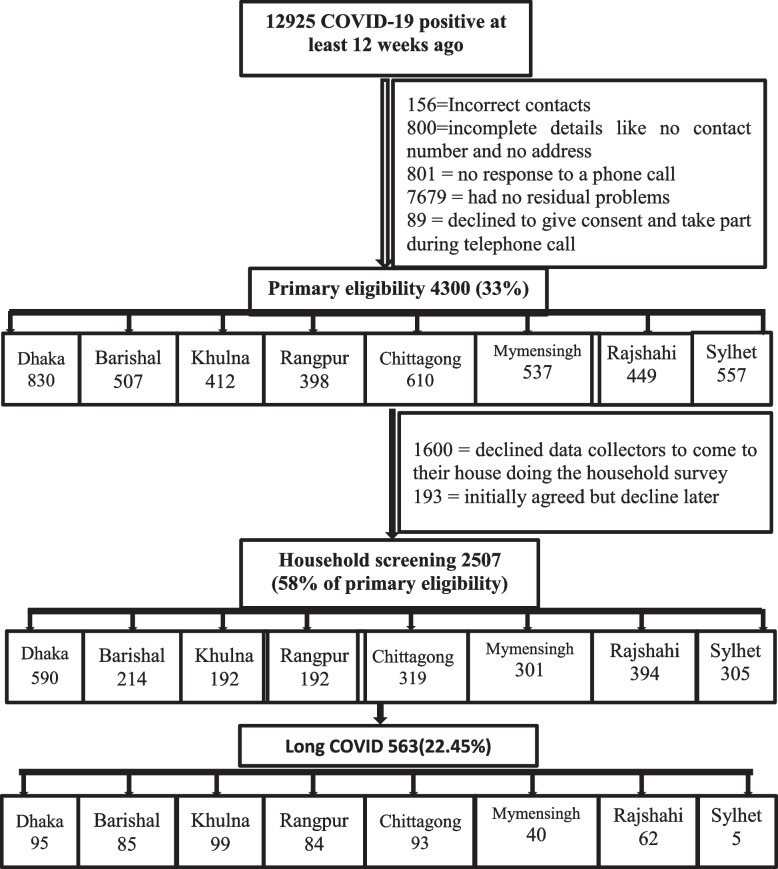
STROBE flow diagram

To enhance the study rigour, we followed the strengthening of the reporting of observational studies in epidemiology (STROBE) guideline for the cross-sectional study (Fig. 1). Primarily we made phone call for the confirmation of household screening in eight administrative divisions. Then we had collected the data through face to face interview. Semi-structured questionnaire used for data collection and it was properly documented. The data was collected by Bachelor of Physiotherapy students who volunteered for the study and were trained for the data collection and diagnosis of long COVID according to WHO Working Group criteria.

### Tools

The sociodemographic and COVID-19-related questionnaire was self-administrated. We utilised the C19-YRS to screen for the Long COVID symptoms [21]. The C19-YRS was a 22-item questionnaire with a binary response option (Yes/ No). The symptom severity was rated on a scale of 0–10, with 0 indicating no symptoms and 10 denoting an extremely severe level. The questionnaire was further categorised into sub-scales: symptom severity score (0–100), functional disability score (0–50), additional symptoms score (0–60), and overall health score (0–10). The C19-YRS was a valid and reliable tool for diagnosing symptom responses for long COVID [2]. The level and intensity of pain, including duration, were diagnosed according to C19-YRS. The questionnaire followed a forward and reverse translation procedure to assure standard consistency. Its Cronbach alpha score was 0.879. The WHOQOL-BREF scale was used to measure QoL in pain-full Long COVID. WHOQOL-BREF was the short version of the WHOQOL-100 containing 26 items addressing four QoL domains: physical health (7 items), psychological health (6 items), social relationships (3 items), and environment (8 items) [22]. Additionally, it was designed to be sensitive to cultural differences. This scale was a valid and reliable tool, and the coefficient value for our questionnaire was 0.716, which was the maximum accepted threshold of a coefficient value of 0.70 [23]. In this study, the measurement of QoL demonstrated high internal consistency, with a Cronbach alpha value of 0.910.

### Data analysis

The data was analysed using the Statistical Package for Social Sciences (IBM SPSS Statistics 20.0). The normality test of the data was performed using the Kolmogorov–Smirnov test and Shapiro–Wilk test with a *P*-value of more than 0.05. Descriptive analysis was performed for sociodemographic variables, health-related variables, and the clinical variables of the respondents, with mean and standard deviation for parametric data and median with interquartile range, frequency and percentage for discrete and non-parametric data. The associations were presented with pain symptoms as the dependent variables through the chi-square test, Fisher’s Exact Test as appropriate. Binary logistic regression was employed the painful symptoms category as dependent and WHOQOL-BREF and C19-YRS score as independent variables. Both adjusted and unadjusted odd ratio (OR) were calculated. The relationship of parametric variables was determined through Pearson correlation. The alpha value was set to *P* < 0.05.

## Results

### Long COVID distribution

In the study, 22.45% (563 out of 2507) of participants reported experiencing Long COVID symptoms, while the remaining 77.54% (1944) were asymptomatic. The participants had an average age of 37.1 ± 13.6 years, with 65.2% falling between the ages of 18 to 40. Among those who experienced Long COVID, 64.3% (362) were in this age group. Males comprised the majority of Long COVID cases at 61.6% (347), while females accounted for 38.4% (216). The majority of Long COVID survivors were married (78.3% or 441), service holders (36.4% or 205), graduates (42.6% or 240), had A (+Ve) blood group (23.5% or 132), were B (+Ve) positive blood group (35.9% or 202), and lived in urban areas (54.3% or 306). Most respondents belonged to middle-income families (62.1% or 350).

According to the findings, the average duration of long COVID was 195.43 ± 63.5 days, with 35.2% of participants experiencing moderate symptoms and 25.7% reporting severe symptoms. Of all the participants, 63.4% received their second vaccine dose, and 21.9% received a booster. Among those with long COVID, 59.3% had received their second dose, 11.2% had only received their first, and 3.9% had not been vaccinated at all. Additionally, 23.2% of participants had diabetes as a major comorbidity, while 28.9% had hypertension. Other comorbidities included osteoarthritis for 6.9% and back pain for 18%. Significant differences were observed between the asymptomatic and long COVID groups in terms of marital status (*P* < 0.01), educational status (*P* < 0.01), family income (*P* < 0.001), vaccination status (*P* < .001), and comorbidity (*P* < .05). Please see Table 1 for a detailed breakdown of the study participants’ demographic profile.

**Table 1:** Long COVID distribution according to demography

Variables	Sub-category	ALL (*N* = 2507)		Asymptomatic (*N* = 1944; 77.54%)		Long COVID (*N* = 563; 22.45%)		*P*
**Age** ^**a**^	Mean ± SD	37.1	±13.6	37.1	±13.7	**37.4**	**±13.5**	0.770
**Age Category** ^**b**^	18–40 Years	1634	(65.2%)	1272	(65.4%)	**362**	**(64.3%)**	0.730
	41–60 Years	719	(28.7%)	548	(28.2%)	**171**	**(30.4%)**	
	> 60 Years	154	(6.1%)	124	(6.4%)	**30**	**(5.3%)**	
**Gender** ^**b**^	Male	1530	(61%)	1183	(60.9%)	**347**	**(61.6%)**	0.760
	Female	977	(39%)	761	(39.1%)	**216**	**(38.4%)**	
**Marital Status**^**b**^	Married	1859	(74.2%)	1418	(73%)	**441**	**(78.3%)**	0.009^**^
	Unmarried	623	(24.9%)	503	(25.8%)	**120**	**(21.3%)**	
	Widow	22	(0.9%)	20	(1%)	**2**	**(0.4%)**	
	Divorce	3	(0.1%)	3	(0.2%)	**0**	**(0%)**	
**Occupation** ^**b**^	Day labor	165	(6.6%)	119	(6.1%)	**46**	**(8.2%)**	0.060
	Service holder	894	(35.7%)	689	(35.4%)	**205**	**(36.4%)**	
	Health Professionals	146	(5.8%)	125	(6.4%)	**21**	**(3.7%)**	
	Law enforcement	128	(5.1%)	86	(4.4%)	**42**	**(7.5%)**	
	Housewife	403	(16.1%)	305	(15.7%)	**98**	**(17.4%)**	
	Student	463	(18.5%)	374	(19.2%)	**89**	**(15.8%)**	
	Unemployed	92	(3.7%)	72	(3.7%)	**20**	**(3.6%)**	
	Businessman	216	(8.6%)	174	(8.9%)	**42**	**(7.5%)**	
**Educational Status** ^**b**^	No formal education	52	(2.1%)	38	(1.95%)	**14**	**(2.5%)**	0.010**
	Primary education	236	(9.4%)	174	(8.95%)	**62**	**(11%)**	
	Secondary education	409	(16.3%)	309	(15.9%)	**100**	**(17.8%)**	
	Higher secondary education	642	(25.6%)	495	(25.5%)	**147**	**(26%)**	
	Bachelor or above	1168	(46.6%)	928	(47.7%)	**240**	**(42.6%)**	
**Blood Group** ^**b**^	A+	628	(25%)	496	(25.5%)	**132**	**(23.5%)**	0.970
	A-	36	(1.4%)	31	(1.6%)	**5**	**(0.9%)**	
	B+	837	(33.4%)	635	(32.6%)	**202**	**(35.9%)**	
	B-	62	(2.5%)	42	(2.2%)	**20**	**(3.6%)**	
	AB+	315	(12.6%)	243	(12.5%)	**74**	**(13.2%)**	
	AB-	23	(0.9%)	19	(1%)	**4**	**(0.7%)**	
	O+	566	(22.6%)	448	(23%)	**118**	**(21%)**	
	O-	39	(1.6%)	32	(1.6%)	**7**	**(1.2%)**	
**Family** ^**b**^	Nuclear Family	1180	(47.1%)	931	(47.9%)	**249**	**(44.3%)**	0.130
	Extended Family	1327	(52.9%)	1013	(52.1%)	**314**	**(55.7%)**	
**Living Area** ^**b**^	Rural	228	(9.1%)	168	(8.6%)	**60**	**(10.7%)**	0.080
	Semi-urban	848	(33.8%)	651	(33.5%)	**197**	**(35.1%)**	
	Urban	1431	(57.1%)	1125	(57.9%)	**306**	**(54.3%)**	
**Monthly Family Income** ^**b**^	Below the poverty line^§^ < 10,200 (< 85 USD)	1	(0.03%)	0	(0%)	**1**	**(0.2%)**	0.001^***^
	< 35,000 BDT (< 320 USD)	1355	(54.0%)	1005	(51.7%)	**350**	**(62.1%)**	
	> 35,000 BDT (> 320 USD)	1151	(45.9%)	939	(48.3%)	**212**	**(37.7%)**	
**Long COVID Duration** ^**a**^	Mean ± SD	195.43	±63.5			**195.43**	**±63.5**	
**Long COVID severity** ^**b**^	Mild	220	(39.1%)			**220**	**(39.1%)**	
	Moderate	198	(35.2%)			**198**	**(35.2%)**	
	Severe	145	(25.8%)			**145**	**(25.7%)**	
**Vaccination** ^**b**^	Non-Vaccinated	109	(4.3%)	87	(4.5%)	**22**	**(3.9%)**	0.001^***^
	1st dose	259	(10.3%)	196	(10.1%)	**63**	**(11.2%)**	
	2nd dose	1590	(63.4%)	1256	(64.6%)	**334**	**(59.3%)**	
	Booster dose	549	(21.9%)	405	(20.8%)	**144**	**(25.6%)**	
**Co-Morbidity** ^**b**^	Heart Disease	142	(8.5%)	106	(8.4%)	**36**	**(8.9%)**	0.025^*^
	Hypertension	480	(28.9%)	348	(27.7%)	**132**	**(32.5%)**	
	Lung Disease	40	(2.4%)	35	(2.8%)	**5**	**(1.2%)**	
	Diabetes	386	(23.2%)	288	(22.9%)	**98**	**(24.3%)**	
	Ulcer	71	(4.3%)	64	(5.1%)	**7**	**(1.7%)**	
	Kidney	34	(2%)	27	(2.1%)	**7**	**(1.7%)**	
	Liver	13	(0.8%)	11	(0.9%)	**2**	**(0.5%)**	
	Anaemia	17	(1%)	12	(1%)	**5**	**(1.2%)**	
	Cancer	3	(0.2%)	3	(0.2%)	**0**	**(0%)**	
	Depression	37	(2.2%)	25	(2%)	**12**	**(3.0%)**	
	Osteoarthritis	115	(6.9%)	100	(7.9%)	**15**	**(3.7%)**	
	Back Pain	299	(18%)	216	(17.2%)	**83**	**(20.4%)**	
	Rheumatoid Arthritis	26	(1.6%)	23	(1.8%)	**3**	**(0.7%)**	
**Smoking** ^**b**^	Yes	456	(18.2%)	352	(18.1%)	**104**	**(18.5%)**	0.820
	No	2051	(81.8%)	1592	(81.9%)	**459**	**(81.5%)**	
**Modified Brief pain inventory (MBPI)** ^**a**^	Severity	8.22	±4.009			**8.22**	**±4.009**	
	Interference	9.03	±7.661			**9.03**	**±7.661**	
	Total score	17.23	±10.515			**17.23**	**±10.515**	
**WHOQOL-BREF** ^**a**^	Physical health	16.82	±6.755	72.06	±12.179	**16.82**	**±6.755**	0.001^***^
	Psychological health	17.43	±6.131	65.95	±10.036	**17.43**	**±6.131**	0.001^***^
	Social relationship	18.55	±6.848	78.27	±12.966	**18.55**	**±6.848**	0.001^***^
	Environmental Health	17.24	±7.317	73.42	±13.630	**17.24**	**±7.317**	0.001^***^
	Self-rating overall perception of QoL(Q1)	3.75	±0.793	3.75	±0.809	**3.76**	**±0.737**	0.824
	Self-satisfaction with their Health(Q2)	3.64	±0.890	3.65	±0.906	**3.63**	**±0.832**	0.769

^a^ One-way ANOVA, ^b^ Friedman’s ANOVA among mild, moderate, and severe cases with significant values (*P*) as < 0.001^***^, < 0.01^**^, and < 0.05^*^

^§^ Household income and expenditure survey HIES 2022 - bbs.portal.gov.bd

### Quality of life

**Fig. 2:**
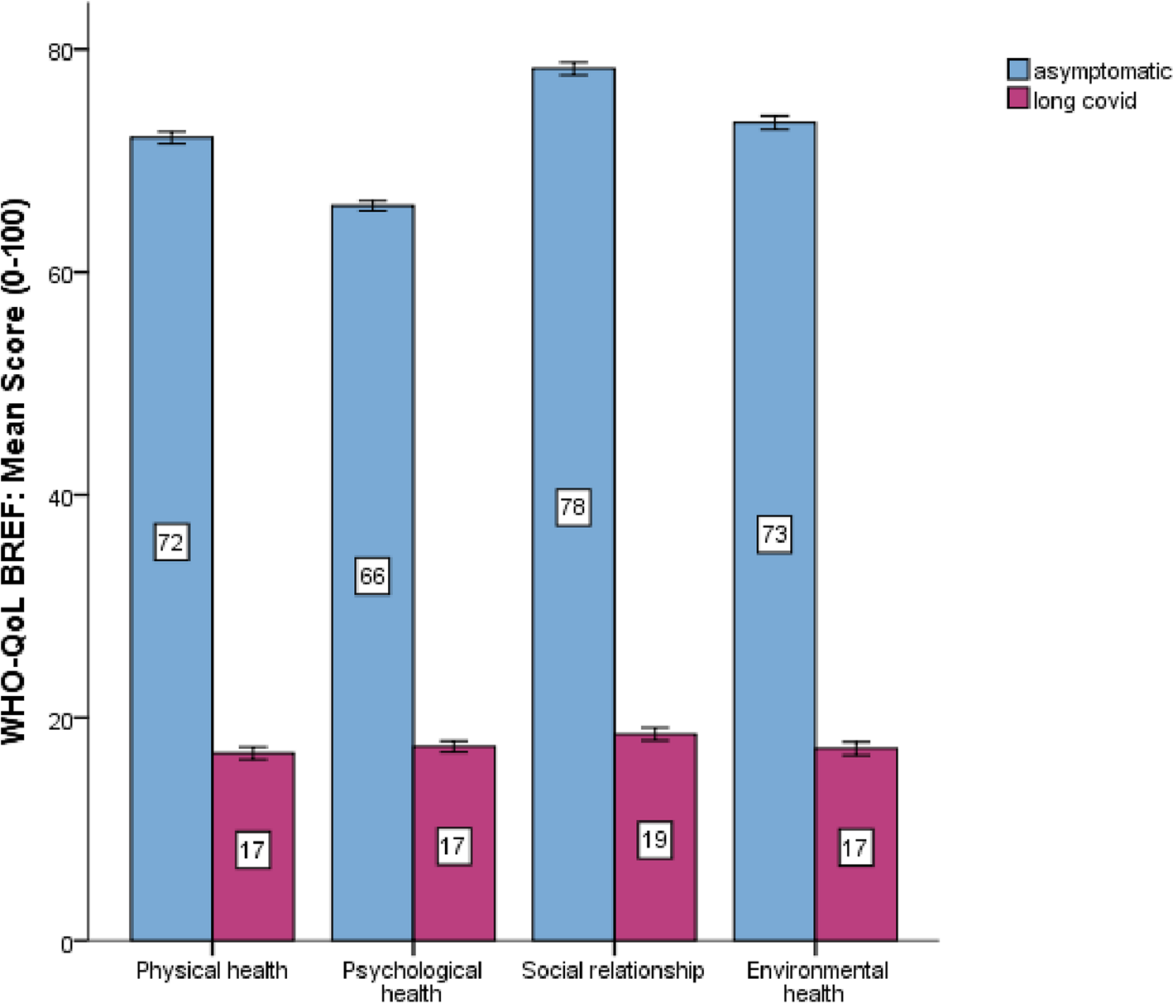
Quality of life status in Long COVID and Asymptomatic cases

The QoL status of the entire study population according to WHOQOL-BREF was as follows: physical health 16.8 ± 6.75, psychological health 17.43 ± 6.13, social relationship 18.5 ± 6.8 and environmental health 17.2 ± 7.31. In long COVID cases, the physical health status was lower than the asymptomatic cases at 16.8 ± 6.7 compared to 72.06 ± 12.1. Similarly, the long COVID cases had significantly lower status in psychological health, social relationships, and environmental health than asymptomatic cases. In psychological health, long COVID cases had 17.43 ± 6.13 compared to asymptomatic cases at 65.95 ± 10.0, and in social relationship, long COVID cases had 18.55 ± 6.8 compared to 78.27 ± 12.9 and in environmental health, 17.24 ± 7.3 compared to 73.42 ± 1.3. In all the domains of QoL, long COVID patients had significantly lower status of QoL than asymptomatic cases *P* < 0.001 (Table 1). The QoL status in long COVID and non-long COVID cases presented in Fig. 2, and in Table 2 the QoL status in painful and non-painful cases were illustrated, and in Table 3, the logistic regression analysis in multiple long COVID symptoms were presented.

**Table 2:** Pain symptoms as Long COVID

Symptoms	Population Proportion in %	95% CI in % of population
Chest Pain	2.4%	1.8–3.1
Joint pain	2.8%	2.2–3.5
Muscle pain	3.1%	2.4–3.8
Headache	3.1%	2.4–3.8
Abdominal pain	0.3%	0.1–0.5

**Table 3:** The pain symptoms associated with Long COVID

Correlations								
		Physical health	Psychological health	Social relationship	Environmental Health	Long COVID Duration	Long COVID symptoms number	Pain score
Physical health	Pearson Correlation	1	0.837^a^	0.878^a^	0.879^a^	−0.843^a^	−0.568^a^	−0.783^a^
	Sig.		0.000	0.000	0.000	0.000	0.000	0.000
	N	2507	2507	2507	2507	2507	2507	2507
Psychological health	Pearson Correlation	0.837^a^	1	0.829^a^	0.856^a^	−0.853^a^	−0.573^a^	−0.804^a^
	Sig.	0.000		0.000	0.000	0.000	0.000	0.000
	N	2507	2507	2507	2507	2507	2507	2507
Social relationship	Pearson Correlation	0.878^a^	0.829^a^	1	0.840^a^	−0.849^a^	− 0.569^a^	− 0.802^a^
	Sig.	0.000	0.000		0.000	0.000	0.000	0.000
	N	2507	2507	2507	2507	2507	2507	2507
Environmental Health	Pearson Correlation	0.879^a^	0.856^a^	0.840^a^	1	−0.830^a^	−0.560^a^	−0.765^a^
	Sig.	0.000	0.000	0.000		0.000	0.000	0.000
	N	2507	2507	2507	2507	2507	2507	2507
Long COVID duration	Pearson Correlation	−0.843^a^	−0.853^a^	−0.849^a^	−0.830^a^	1	0.686^a^	0.834^a^
	Sig.	0.000	0.000	0.000	0.000		0.000	0.000
	N	2507	2507	2507	2507	2507	2507	2507
Long COVID symptoms number	Pearson Correlation	−0.568^a^	−0.573^a^	−0.569^a^	−0.560^a^	0.686^a^	1	0.564^a^
	Sig.	0.000	0.000	0.000	0.000	0.000		0.000
	N	2507	2507	2507	2507	2507	2507	2507
Pain score	Pearson Correlation	−0.783^a^	−0.804^a^	−0.802^a^	−0.765^a^	0.834^a^	0.564^a^	1
	Sig.	0.000	0.000	0.000	0.000	0.000	0.000	0.000
	N	2507	2507	2507	2507	2507	2507	2507

^a^Correlation is significant at the 0.01 level

### Pain symptoms as long COVID

Among those diagnosed with long COVID, five types of pain were identified: chest pain 2.4% (95%CI; 1.8–3.1%), joint pain 2.8% (95%CI; 2.2–2.35%), muscle pain 3.1% (95% CI; 2.4–3.8%), headache 3.1% (95% CI; 2.4–3.8%), and abdominal pain 0.3% (95% CI; 0.01–0.5%) (Table 2). Table 3 depicts the pain symptoms associated with Long COVID. Among the Long COVID people, the severity of the pain in brief pain inventory was 8.22 ± 4.09 for severe pain and 9.03 ± 7.661 for pain interference.

### Relationships of quality of life and pain

**Fig. 3:**
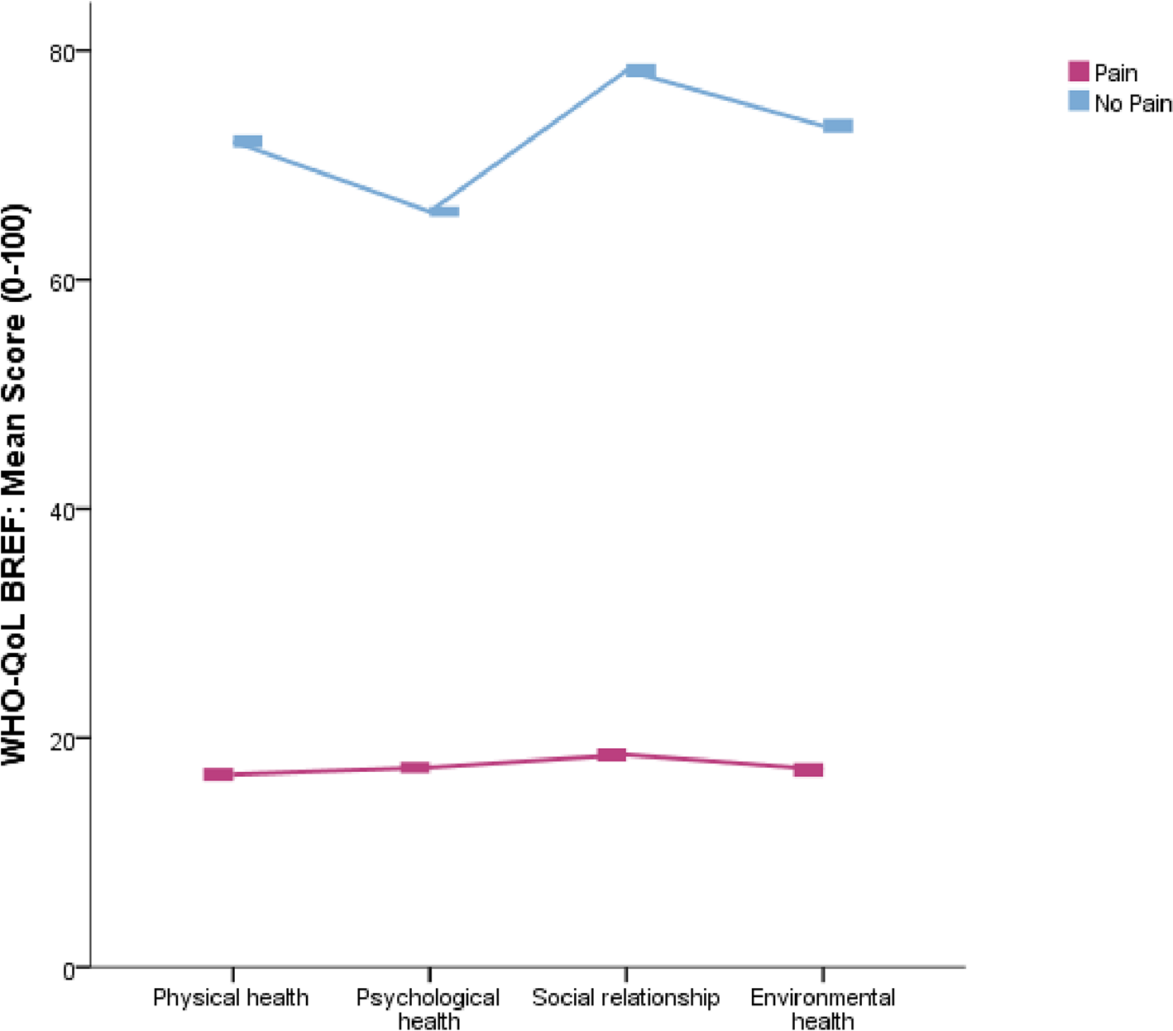
Quality of life status in respondents with or without painful symptoms

**Fig. 4:**
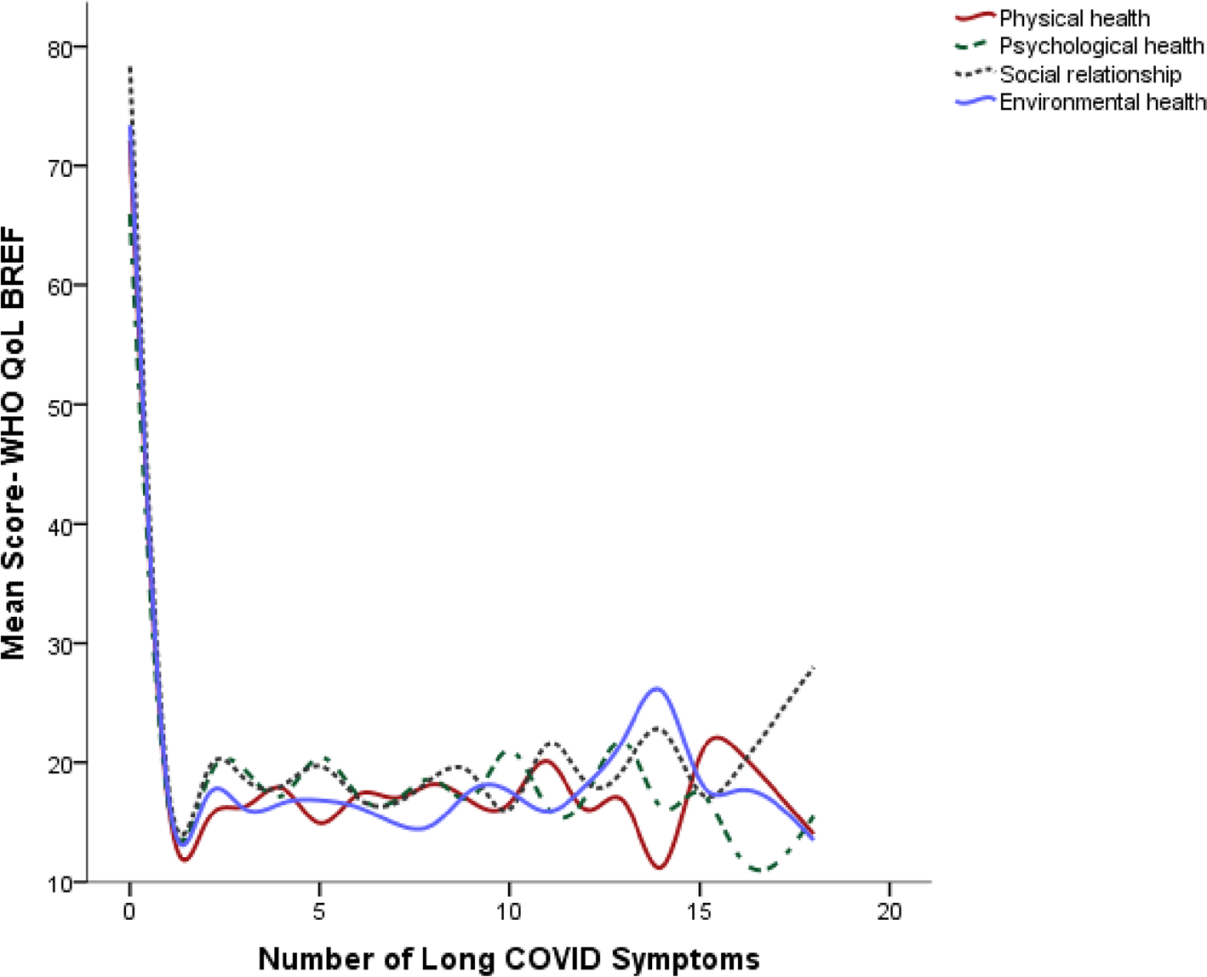
Quality of life status in relation to number of Long COVID symptoms

A significant inverse relationship had found between the physical health status of QoL and long COVID duration − 0.843 (*P* < 0.01), long COVID number of symptoms − 0.568 (*P* < 0.01), and pain scores − 0.783 (*P* < 0.01). Similarly, psychological health had an inverse relationship with long COVID duration − 0.853 (*P* < 0.01), long COVID number of symptoms − 0.573 (*P* < 0.01), and pain scores − 0.804 (*P* < 0.01). In QoL, social relationships had a significantly associated reverse relationship with long COVID duration − 0.849 (*P* < 0.01), long COVID number of symptoms − 0.569 (*P* < 0.01) and pain score − 0.802 (*P* < 0.01). The environmental health domain of QoL also had a significant inverse relationship with long COVID duration, long COVID symptom number and pain score *P* < 0.01 (Table 3). Figure 2 showed QoL status in painful and asymptomatic cases, Fig. 3 showed quality of life status in painful and no-pain cases, and Fig. 4 showed quality of life status in multiple long COVID symptoms.

### Predictors of pain

In binary logistic regression, chest pain was predicted as adjusted odds ratio 0.15 (95% CI; 0.03–0.78) *P* < 0.05, duration of long COVID *P* < 0.001, long COVID symptoms severity *P* < 0.001 and people had poor psychological health status in quality-of-life *P* < 0.01. The predictors of joint pain as long COVID was predicted to people aged 41 to 60 years unadjusted odd ratio (OR) 2.31 (95% CI; 1–5.31) *P* < 0.05, adjusted OR 0.39 (95% CI; 0.20–0.76) *P* < 0.01 (Table 4). Other predictors of joint pain included long COVID duration, number of symptoms of long COVID, physical health, psychological health, social relationship and environmental health *P* < 0.001. Predictors of muscle pain as long COVID included the participants ages 41 to 60 years adjusted OR 0.49 (95% CI; 0.27–0.92) *P* < 0.05. Smoking adjusted OR 0.38 (95% CI; 0.16–0.87) *P* < 0.05. Other predictors of muscle pain were similar to joint pain with *P* < 0.001. Predictors of headache included smoking adjusted OR 0.32 (95% CI; 0.03–0.78) *P* < 0.01. Vaccination with booster dose unadjusted OR 1.67 (95% CI; 1.01–2.76) *P* < 0.05. Number of Long COVID symptoms and *P* < 0.001 and physical health *P* < 0.05. Predictors of abdominal pain as Long COVID was predicted by the multiple number of Long COVID symptoms of a respondent unadjusted OR 0.60 (95% CI; 0.50–0.71) *P* < 0.001, adjusted OR 0.45 (95% CI; 0.30–0.69) *P* < 0.001 (Table 4).

**Table 4:** Logistic regression analysis in multiple long COVID symptoms

Independent Variable	Unadjusted OR (95% CI)	*P*-value	Adjusted OR (95% CI)	*P*-value
**Chest pain**				
Occupation				
Day labor	Reff.		Reff.	
Service holder	1.37 (0.55–3.40)	0.500	0.82 (0.26–2.58)	0.732
Health Professionals	0.88 (0.28–2.79)	0.829	0.15 (0.03–0.78)	0.024*
Law enforcement	6.09 (0.00–7.31)	0.996	3.18 (0.00–5.37)	0.995
Housewife	1.35 (0.49–3.69)	0.566	0.79 (0.18–3.46)	0.756
Student	2.87 (0.91–9.04)	0.071	1.47 (0.31–6.97)	0.628
Unemployed	1.12 (0.27–4.59)	0.875	0.49 (0.08–2.86)	0.427
Businessman	1.59 (0.48–5.31)	0.449	0.93 (0.23–3.85)	0.925
Long COVID Duration	0.98 (0.98–0.99)	0.0001***	1.00 (1.00–1.01)	0.009**
Long COVID symptoms	0.69 (0.66–0.73)	0.0001***	0.76 (0.71–0.82)	0.001***
Physical Health	1.08 (1.06–1.10)	0.0001***	1.04 (0.99–1.04)	0.060
Psychological Health	1.10 (1.07–1.12)	0.0001***	1.07 (1.02–1.12)	0.004**
Social Relationship	1.07 (1.05–1.09)	0.0001***	1.02 (0.98–1.07)	0.289
Environmental Health	1.07 (1.05–1.09)	0.0001***	0.99 (0.95–1.03)	0.586
**Joint Pain**				
Age				
18–40 Years	Reff.		Reff.	
41–60 Years	2.31(1.00–5.31)	0.049*	0.39 (0.20–0.76)	0.006**
> 60 Years	1.06 (0.46–2.45)	0.897	0.34 (0.11–1.08)	0.068
Long COVID Duration	0.986 (0.98–0.99)	0.0001***	1.00 (0.00–1.00)	0.440
Long COVID symptoms	0.58 (0.54–0.63)	0.0001***	0.65 (0.59–0.71)	0.0001
Physical Health	1.08 (1.06–1.10)	0.0001***	1.02 (0.98–1.06)	0.382
Psychological Health	1.09 (1.07–1.11)	0.0001***	1.03 (0.98–1.08)	0.233
Social Relationship	1.07 (1.05–1.09)	0.0001***	1.01 (0.96–1.05)	0.814
Environmental Health	1.08 (1.06–1.10)	0.0001***	1.03 (0.98–1.07)	0.234
**Muscle pain**				
Age				
18–40 Years	Reff.		Reff.	
41–60 Years	1.37 (0.53–3.56)	0.512	0.49 (0.27–0.92)	0.026*
> 60 Years	0.64 (0.25–1.65)	0.353	0.85 (0.24–2.98)	0.759
Smoking				
No	Reff.		Reff.	
Yes	0.64 (0.33–1.24)	0.184	0.38 (0.16–0.87)	0.022*
Long COVID Duration	0.98 (0.98–0.99)	0.0001***	1.00 (0.99–1.00)	0.369
Long COVID symptoms	0.52 (0.47–0.56)	0.0001***	0.56 (0.51–0.63)	0.0001***
Physical Health	1.08 (1.06–1.10)	0.0001***	1.03 (0.99–1.03)	0.180
Psychological Health	1.09 (1.07–1.11)	0.0001***	1.02 (0.97–1.07)	0.438
Social Relationship	1.07 (1.06–1.09)	0.0001***	1.00 (0.96–1.05)	0.973
Environmental Health	1.08 (1.06–1.10)	0.0001***	1.04 (0.99–1.09)	0.112
**Headache**				
Smoking				
No	Reff.		Reff.	
Yes	1.98 (0.95–4.14)	0.070	0.32 (0.13–0.78)	0.010**
COVID vaccine				
Non-Vaccinated	Reff.		Reff.	
1st dose	5.15 (0.69–38.44)	0.110	0.52 (0.04–1.06)	0.599
2nd dose	1.49 (0.67–3.37)	0.329	0.99 (0.10–9.40)	0.989
Booster dose	1.67 (1.01–2.76)	0.040*	0.40 (0.04–4.14)	0.444
Long COVID Duration	0.98 (0.98–0.99)	0.0001***	1.00 (0.99–1.00)	0.457
Long COVID symptoms	0.51 (0.47–0.56)	0.0001***	0.55 (0.49–0.62)	0.001***
Physical Health	1.08 (1.06–1.10)	0.0001***	1.04 (0.99–1.09)	0.042*
Psychological Health	1.09 (1.07–1.11)	0.0001***	0.99 (0.95–1.04)	0.774
Social Relationship	1.08 (1.06–1.10)	0.0001***	1.04 (0.99–1.09)	0.157
Environmental Health	1.08 (1.06–1.09)	0.0001***	1.01 (0.96–1.06)	0.724
**Abdominal Pain**				
Long COVID Duration	0.98 (0.98–0.99)	0.0001***	1.10 (0.99–1.23)	0.207
Long COVID symptoms	0.60 (0.50–0.71)	0.0001***	0.45 (0.30–0.69)	0.001***
Physical Health	1.07 (1.02–1.12)	0.003**	1.01 (0.89–1.14)	0.837
Psychological Health	1.08 (1.02–1.15)	0.004**	1.06 (0.90–1.25)	0.460
Social Relationship	1.07 (1.02–1.12)	0.005**	1.12 (0.96–1.29)	0.159
Environmental Health	1.06 (1.02–1.11)	0.002**	0.89 (0.76–1.06)	0.194

*OR* ODD Ratio, *CI* Confidence Interval, ^*^P < 0.05, ^**^P < 0.01, ^***^P < 0.001, Binary Logistic

## Discussion

This study aimed to assess the prevalence and spectrum of pain symptoms in long COVID survivors in Bangladesh and to discern the association between these symptoms and their QoL. We identified five principal categories of pain: chest pain, joint pain, muscle pain, headache, and abdominal pain, with prevalence rates between 0.03–3.1%. Our findings also confirmed a compromised QoL across all the domains of the WHOQOL-BREF when compared to asymptomatic participants. There was a strong and inverse relationship between painful symptoms, duration of long COVID, and number of long COVID symptoms in all the domains of QoL majors. This study found that painful and multiple symptoms with longer duration affect an individual’s QoL who are survivors of long COVID.

The study explored five types of pain for individuals with long COVID in Bangladesh, and the prevalence of pain ranged from 0.3% (headache) to 3.1% (muscle pain). In Pakistan, myalgia and arthralgia was reported in approximately 63.63% of males and 36.36% of females [24]. In comparison, these percentages were much lower in Sweden (2.2%) [25], China (3 to 7%), [26], and Norway (arthralgia at 9% and myalgia at 8.5%) [27], In Bangladesh [28] 1.2% myalgia and 4.8% arthralgia. The occurrence of distressing symptoms may vary based on the diagnostic standards applied. Research utilising the WHO Working Group classification to diagnose such symptoms indicates a prevalence of less than 5%, whereas studies considering symptoms appearing after 4 weeks of COVID-19 indicate a higher occurrence. The overall studies WHO counted painful symptoms after 12 weeks of SARS-COV-2, and the symptoms persisted for 2 months had myalgia ranging from .6 to 3% [25–28] and arthralgia 2.8 to 3.6% [26, 28]. Our study filled up the research gap on the actual population prevalence and characterisation of painful symptoms as long COVID.

According to the findings of this study, there exists a distinct correlation between the frequency and duration of painful symptoms (chest pain, joint pain, muscle pain, headache, abdominal pain) of long COVID cases and a reduction in quality of life. To be more precise, those who endure painful symptoms or numerous long COVID symptoms over extended periods often encounter a marked decline in their physical health, psychological well-being, social connections, and environmental factors. These domains were evaluated using the WHOQOL-BREF scale. Literature was available exploring impaired health-related QoL for long COVID after mild to moderate COVID-19 [15]. Also, another research [16] explores that long COVID people generally had declined QoL compared to the healthy control group. Moreover, a Bangladeshi study also found that people with long COVID had significantly lower QoL measures and a mixed level of coping strategies adopted after the fast surge of the COVID-19 pandemic [21].

Our study suggests certain demographic and behavioural predictors that may influence pain prevalence in long COVID survivors. Occupationally, health professionals emerged as a vulnerable group. Other factors include the female gender, individuals aged between 41 and 60, smoking habits, multiple long COVID symptoms, and a pre-existing poor health status. A Turkish study found that female survivors are more likely to have myalgia and joint pain [6]. Similarly, an American study indicates a higher propensity for females to experience myalgia [29]. In contrast, an Egyptian study identified depression, moderate to severe antibiotic use in COVID-19, and poor health as predictors of arthralgia and myalgia in long COVID patients [30]. Interestingly, a study conducted in Bangladesh observed a rise in psychological and environmental health scores as individuals age [18]. Moreover, certain chronic illnesses significantly reduce the overall quality of life. A study had revealed that chronic diseases have a negative impact on all aspects of QoL [19]. These significant predictors contribute to the development of widespread types of pain in five general categories: chest pain, joint pain, muscle pain, headache, and abdominal pain.

### Strength and limitation

The study followed the structure reporting of observational studies STROBE guidelines and adhered to Helsinki guidelines to determine the painful symptoms of the long COVID survivors in Bangladesh. The study had some limitations but did not significantly affect the results. A lack of data might be an undesirable effect on the generalizability of the findings. This study employed a cross-sectional design, however, to adequately assess the long-term painful symptoms of Long COVID, longitudinal surveillance is necessary. We suggested that future research incorporate a larger dataset to examine the presence of persistent and painful symptoms associated with long COVID, as well as their impact on quality of life. In addition to obtaining more detailed ideas, collecting data in long COVID is crucial. Increasing the number of participants in the WHOQOL-BREF domain gives a better understanding of the issues. The inter-cluster correlation of the study was at a minimum level. So, the rate of responses in the different divisions of Bangladesh was uneven, and there were discrepancies. We managed this limitation by ensuring the minimum sample size required in each division. The study addressed a significant research gap in the characterisation, and estimation of painful symptoms from long COVID and predicting the risk factors.

## Recommendation

We recommend future studies on observing the long-term consequences of pain symptoms for the long COVID survivors and the outcome of physiotherapy and rehabilitation interventions within the scope of rehabilitation for managing painful symptoms of long COVID.

## Conclusion

Bangladeshi people with long COVID have five types of pain symptoms, and the prevalence ranges from 0.3 to 3.1%, where most individuals have myalgia and arthralgia. Painful and multiple symptoms were associated with a degraded QoL for the long COVID survivors and middle-aged people. Individuals with multiple long COVID symptoms and longer duration of long COVID, including females, are the most vulnerable people with painful episodes of long COVID in Bangladesh.

### Supplementary Information

**Supplementary Material 1.**

## Data Availability

The data was available in online database (Mendeley Data) site. [Kabir, Md Feroz (2023), “Long COVID Data Set”, Mendeley Data, V1, doi: 10.17632/rzcfrpsrm6.1].

## Abbreviations

LC: Long COVID symptoms
QoL: Quality of life
WHO: World health organization
C19-YRS: COVID-19 yorkshire rehabilitation scale
CI: Confidential interval
